# Minimal RNA self-reproduction discovered from a random pool of oligomers†

**DOI:** 10.1039/d3sc01940c

**Published:** 2023-06-20

**Authors:** Ryo Mizuuchi, Norikazu Ichihashi

**Affiliations:** a Department of Electrical Engineering and Bioscience, Faculty of Science and Engineering, Waseda University Shinjuku Tokyo 162-8480 Japan mizuuchi@waseda.jp; b JST, FOREST Kawaguchi Saitama 332-0012 Japan; c Komaba Institute for Science, The University of Tokyo Meguro Tokyo 153-8902 Japan; d Department of Life Science, Graduate School of Arts and Science, The University of Tokyo Meguro Tokyo 153-8902 Japan; e Universal Biology Institute, The University of Tokyo Meguro Tokyo 153-8902 Japan

## Abstract

The emergence of RNA self-reproduction from prebiotic components would have been crucial in developing a genetic system during the origins of life. However, all known self-reproducing RNA molecules are complex ribozymes, and how they could have arisen from abiotic materials remains unclear. Therefore, it has been proposed that the first self-reproducing RNA may have been short oligomers that assemble their components as templates. Here, we sought such minimal RNA self-reproduction in prebiotically accessible short random RNA pools that undergo spontaneous ligation and recombination. By examining enriched RNA families with common motifs, we identified a 20-nucleotide (nt) RNA variant that self-reproduces *via* template-directed ligation of two 10 nt oligonucleotides. The RNA oligomer contains a 2′–5′ phosphodiester bond, which typically forms during prebiotically plausible RNA synthesis. This non-canonical linkage helps prevent the formation of inactive complexes between self-complementary oligomers while decreasing the ligation efficiency. The system appears to possess an autocatalytic property consistent with exponential self-reproduction despite the limitation of forming a ternary complex of the template and two substrates, similar to the behavior of a much larger ligase ribozyme. Such a minimal, ribozyme-independent RNA self-reproduction may represent the first step in the emergence of an RNA-based genetic system from primordial components. Simultaneously, our examination of random RNA pools highlights the likelihood that complex species interactions were necessary to initiate RNA reproduction.

## Abstract 2

An RNA oligomer that self-reproduces *via* prebiotically plausible chemistry was identified by examining short random RNA pools. The oligomer exhibits a potential for exponential self-reproduction, similar to the behavior of much larger ribozymes.

## Graphical abstract

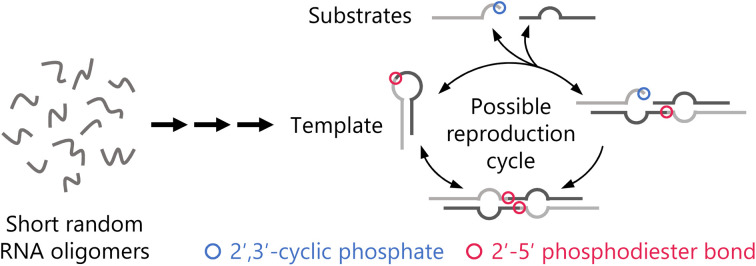

## Introduction

The first genetic system before the emergence of life may have been based on RNA, because RNA can simultaneously carry genetic information and catalyze chemical reactions.^1,2^ This “RNA World” hypothesis is supported by the observation that all genetically encoded proteins are synthesized by RNA in the ribosome.^3^ A crucial aim in the quest for an RNA-based genetic system is to find self-reproducing RNA molecules.^4–6^ A potential mechanism for RNA reproduction is template-directed polymerization of nucleotides, *i.e*., replication, as observed in extant life. However, despite significant progress in improving non-enzymatic or ribozyme-catalyzed RNA polymerization,^7–10^ the self-replication of these systems remains challenging. Previous studies have therefore explored alternative, simpler mechanisms for RNA self-reproduction through the assembly of oligonucleotides.^6,11,12^ In view of recent clarification, we use the term “reproduction” to denote RNA copying in general by distinguishing canonical “replication” that follows template-directed polymerization chemistry.^6,12^

RNA reproduction has been demonstrated for ligase and recombinase ribozymes.^13–16^ For example, a ligase ribozyme derived from the R3C ligase ribozyme^17^ catalyzes the joining of two RNA substrates as a template to form a sequence identical to itself.^13^ This ribozyme is the simplest self-reproducing RNA known to date in terms of its length (61 nt) and the number of components (two fragments: 13 and 48 nt). However, the ribozyme is still relatively large and was rationally designed, and it remains unclear how such a ribozyme and its components could have been prevalent in prebiotically accessible RNA mixtures, which were likely dominated by shorter (up to ∼20 nt) and random oligonucleotides.^18,19^ The ligase ribozyme also requires 5′-triphosphate activation, which necessitates an additional set of complex reactions.^20^ Consequently, it has been proposed that the template-based self-reproduction of short RNA molecules independent of complex ribozymes may have emerged first in the RNA World.^21,22^

Self-reproduction of short nucleic acids has been studied mainly using DNA. Previous studies demonstrated the autocatalytic reproduction of chemically modified DNA oligonucleotides through template-directed ligation, although the reproduction was severely hindered by two tightly bound templates (or a template and its identical product after ligation).^23–25^ A recent study employed temperature cycling to overcome such template inhibition in the reproduction of chemically activated DNA.^26^ Despite these efforts with DNA, the self-reproduction of short RNA oligomers is currently missing. Moreover, temperature cycling would be incompatible with RNA because, unlike DNA, RNA easily degrades at high temperature, which is accelerated by divalent metal ions^27,28^ that commonly enhance ribozyme catalysis^28,29^ as well as template-directed RNA synthesis.^30,31^

Template-directed ligation of short RNA is achieved in the laboratory using terminal activation such as with 2′,3′-cyclic phosphate (>p),^30^ which readily forms in prebiotically plausible environments,^32,33^ while recombination occurs directly—or in combination with spontaneous >p formation through hydrolysis of RNA, termed α/α′ mechanisms.^31,34^ Notable are recent studies that demonstrated that pools of short random RNA can undergo diverse intermolecular ligation and recombination, presumably in a templated manner.^31,35^ In these populations, RNA products that form efficiently or that self-amplify are expected to be enriched. Thus, a close examination of the enriched products may lead to the discovery of efficient RNA reproduction *via* template-directed ligation or recombination. The identification of such reproducing RNA would also provide insights into the likelihood of the emergence of self-reproduction out of random chemistry.

In this study, we first examined spontaneous ligation and recombination reactions in pools of short random RNAs and found that they can be detected more quickly than previously demonstrated. We observed the enrichment of RNA families with common motifs in multiple RNA pools. Subsequent analyses of the most enriched products and their variants led us to find a short (20 nt) RNA oligomer that can self-reproduce *via* template-directed ligation of two 10 nt substrates. The RNA contains a 2′–5′ phosphodiester bond, a linkage usually generated during non-enzymatic RNA synthesis.^36–38^ Partly due to the non-canonical linkage, the RNA circumvented its dimerization and displayed a potential for exponential reproduction in an isothermal environment, although the restricted formation of an active complex with the substrates limited the amplification. Its autocatalytic properties and structures are somewhat similar to those of the previously developed self-reproducing ligase ribozyme.^13^ These results demonstrate the first example of minimal RNA self-reproduction independent of a ribozyme and also help understand the dynamics of primordial, random RNA pools.

## Results and discussion

### Incubation of short random RNA pools

**Fig. 1:**
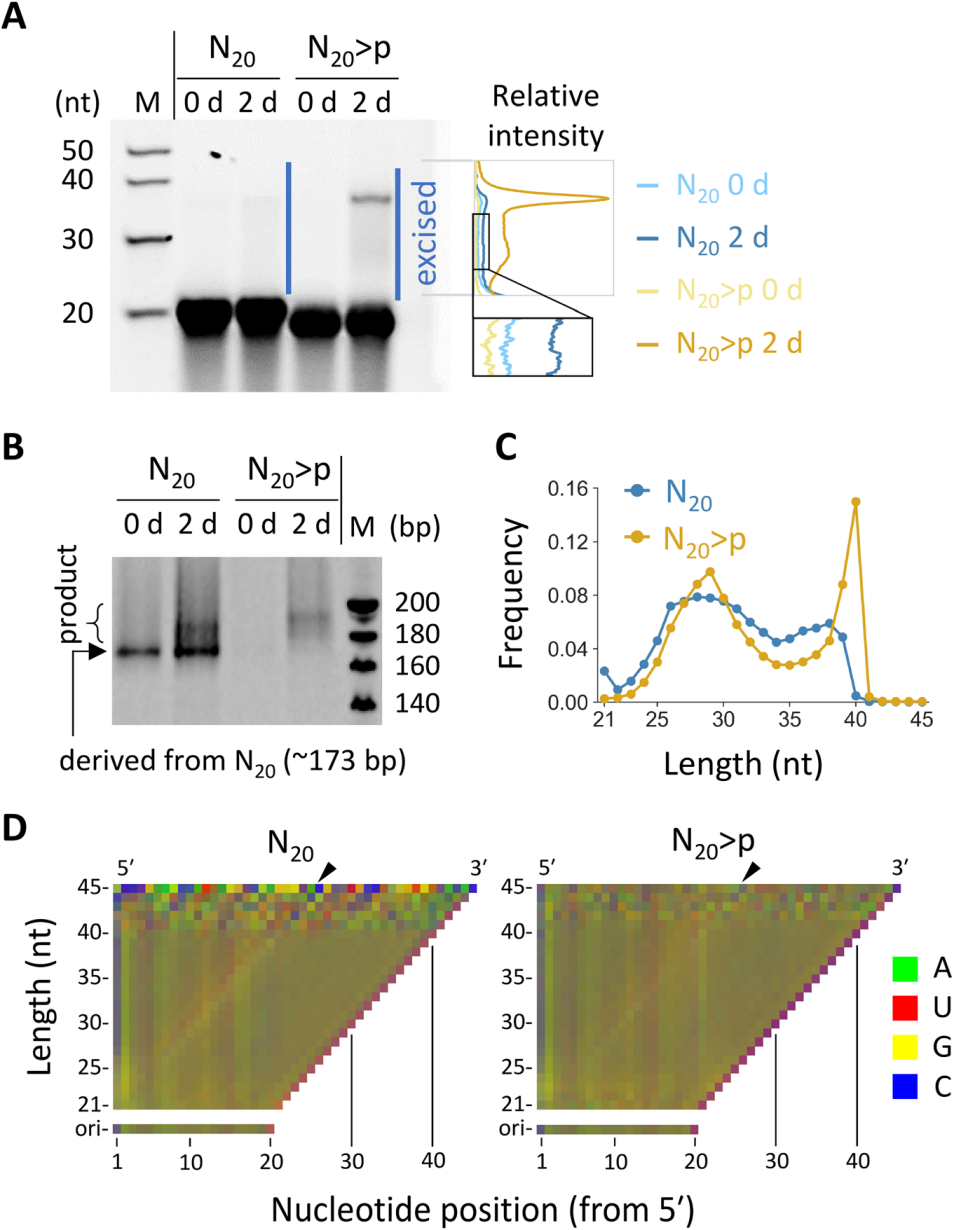
Reactivity of N_20_ and N_20_>p RNA pools. (A) Incubation of 50 μM N_20_ or N_20_>p in 100 mM MgCl_2_ at 22 °C and pH 8.0 for 2 days, analyzed by 20% denaturing PAGE. RNA products (*ca.* 21–45 nt) were excised and subjected to RT-PCR. Note that RNA with >p migrates slightly faster. Relative band intensities of the indicated region are shown on the right. The dependence of band intensity on RNA length was not calibrated. (B) RT-PCR products analyzed by 15% native PAGE. Different PCR cycles were applied to the N_20_ and N_20_>p samples. (C) Length distribution of 21–45 nt products detected multiple times in the HTS analyses. (D) Nucleotide compositions of the products with each length for N_20_ (left) and N_20_>p (right) pools. The frequency of each nucleotide was represented by a linear combination of RGB values as in the previous study.^35^ The compositions of the original 20 nt pools were displayed for comparison. Arrowheads indicate putative ligation junctions.

We investigated reactions in fully random 20 nt RNA (N_20_), which was previously shown to undergo both ligation and recombination if pre-activated with >p.^35^ We prepared N_20_ and N_20_>p pools containing ∼3 × 10^14^ molecules to cover all possible ∼10^12^ sequences of 20 nt with redundancy (∼300 copies). Previous studies detected ligation and recombination in 16–20 nt random RNA pools (5–100 μM) only after incubation for months or longer times in ice.^31,35^ However, we found that, in the presence of high concentration (100 mM) of MgCl_2_, which promotes >p-mediated template-directed ligation and recombination,^30,31^ both N_20_ and N_20_>p pools (50 μM) generated detectable >20 nt products after just a 2 day incubation, as visualized by denaturing polyacrylamide gel electrophoresis (PAGE) (Fig. 1A). Note that degraded fragments in the initial pools may also have contributed to the reactions.

To examine sequences enriched in the random RNA pools, we excised the elongated products (*ca.* 21–45 nt) in both N_20_ and N_20_>p pools from a denaturing polyacrylamide gel and subjected them to RT-PCR and high-throughput sequencing (HTS). The RT-PCR was performed using the SMARTer technology, *via* poly-A tailing and following template switching during reverse transcription. We detected PCR products for both N_20_ and N_20_>p pools only if they were pre-incubated for two days, confirming recombination and ligation during the incubation (Fig. 1B). From the HTS data, we analyzed 374 357 and 412 461 reads of 21–45 nt products that were detected at least twice for the N_20_ and N_20_>p pools, respectively. The majority of the products derived from the N_20_ pool were 24–39 nt (95%) with a sharp drop-off above 39 nt (Fig. 1C), indicating that they were generated primarily by recombination, because a single recombination of two 20 nt RNAs could lead to a 21–39 nt product. On the other hand, the products in the N_20_>p pool were predominantly 24–40 nt (98%) with a sharp peak at 40 nt (Fig. 1C), suggesting that both recombination and ligation operated in the pool. It should be noted that recombination could occur either directly or indirectly *via* ligation on >p of a hydrolyzed RNA.^31^ The nucleotide compositions in the <40 nt products of the random RNA pools displayed a slight enrichment in G at the both sides of a putative ligation junction between a cleaved RNA>p (<20 nt) and a 20-mer (Fig. 1D and S1,† in the direction indicated by black arrowheads), which was more evident in the N_20_ pool than in the N_20_>p pool. The results contrast with the previous studies that incubated random RNAs in ice and without MgCl_2_, where cytosine and/or uracil were particularly enriched as putative phosphate donors.^31,35^ The predicted secondary structures of the products tended to be more stable than those of random sequences of the same sizes and nucleotide compositions (Fig. S2†), consistent with a previous study.^35^

### Identification of enriched RNA families

**Fig. 2:**
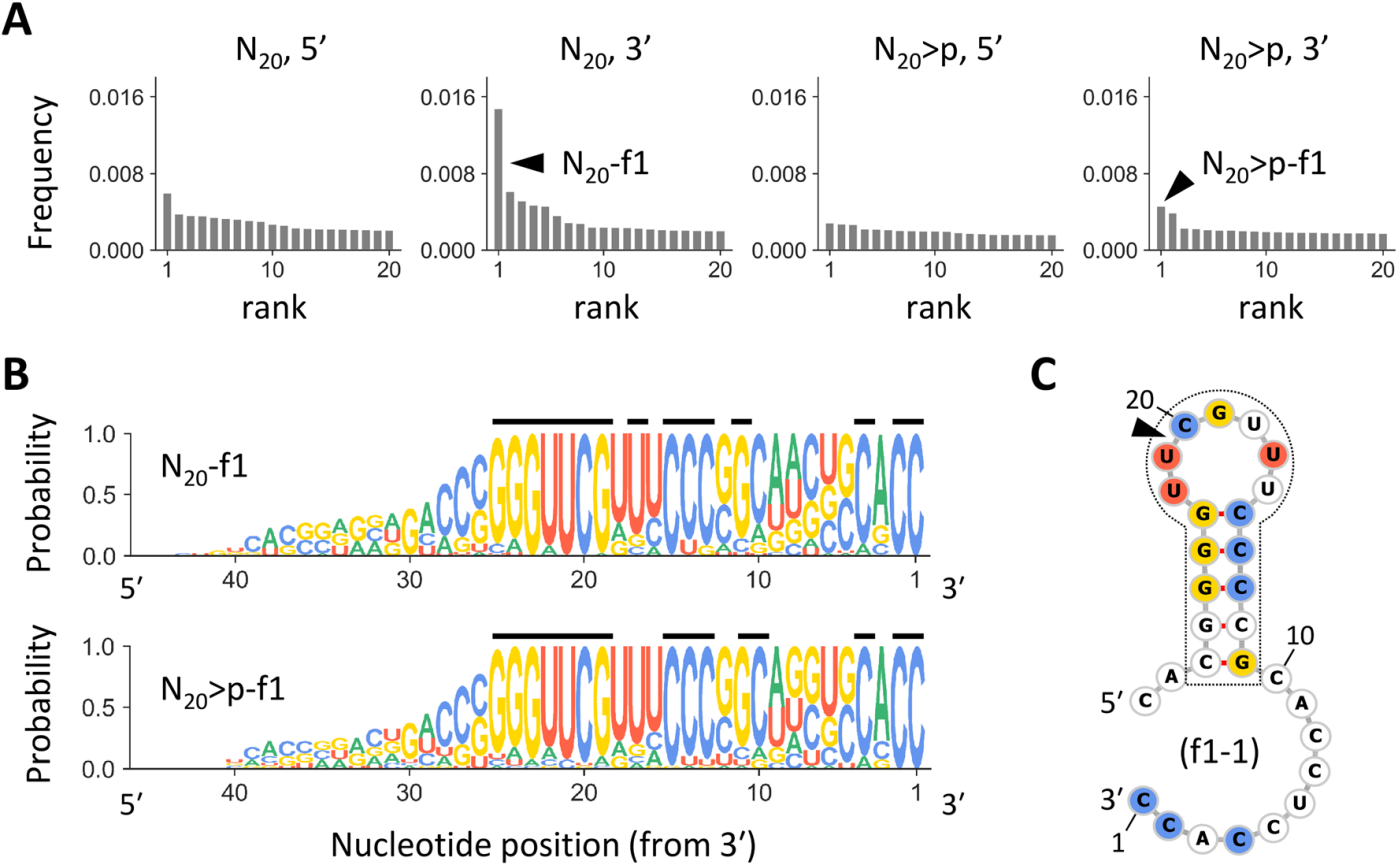
The most enriched RNA families. (A) Frequencies of the most enriched 20 RNA families in analyzed N_20_ or N_20_>p-derived products, sorted in descending order. Each panel represents families constructed based on sequence similarity around 5′ or 3′ terminus for N_20_ or N_20_>p. The arrowheads indicate N_20_-f1 and N_20_>p-f1. (B) Nucleotide compositions in sequences of N_20_-f1 (top) and N_20_>p-f1 (bottom). The sequence logos show the probability of each nucleotide at each position, calculated by ignoring the redundancy of each sequence (Fig. S3†). The black lines above indicate sites where a specific nucleotide is detected with a probability of >0.8. (C) A predicted secondary structure of f1-1, the most abundant sequence in N_20_-f1. Nucleotides detected with a probability of >0.8 are colored according to panel B. The commonly observed stem-loop structure is enclosed in the dotted line. The arrowhead indicates the putative recombination junction.

If the RNA products were synthesized by previously identified ligation or recombination mechanisms,^30,31,34^ 20 nt sequences in the original pools should remain intact at the 5′ or 3′ end of the products, consistent with the enrichment of specific nucleotides at the putative junctions (Fig. 1D and S1†). Thus, we grouped the most abundant 10 000 products from each pool of N_20_ and N_20_>p into families based on sequence similarity around the 5′ or 3′ terminus. Products differ from the most abundant sequence of each family by seven or fewer edits for the 21 nucleotides at each end. When grouping N_20_-derived products by their 3′ ends, we observed a highly enriched family, named N_20_-f1, that comprised ∼1.5% of all analyzed products. This family was 2.4-fold more abundant than the second most enriched family (Fig. 2A). The N_20_-f1 family consists of 93 sequences that were well aligned at the 3′ end (Fig. 2B). More than 80% of them contained common nucleotides at positions 1, 2, 4, 13–15, 17, and 19–25 from the 3′ end (indicated by the black lines), while nucleotides at other positions were relatively random. Likewise, when grouping the N_20_>p-derived products by their 3′ ends, we found an enriched family with a similar set of sequences, N_20_>p-f1 (Fig. 2B). Although N_20_>p-f1 was the most abundant in the pool, the frequency was comparable to other low-rank families and comprised ∼0.6% of the analyzed products (Fig. 2A). 17 sequences were commonly found in both N_20_-f1 (18%) and N_20_>p-f1 (43%). The enrichment of specific families was less clear when grouped by the 5′ end (Fig. 2A). Other high-ranked families are described in Fig. S4;† some of them have similar nucleotide compositions to N_20_-f1 and N_20_>p-f1. We also note that in the same analyses using the synthetic sequences (Fig. S2†), unsurprisingly, the most enriched families represented only ∼0.2% for each set, and their components did not align at all.

RNA sequences in N_20_-f1 and N_20_>p-f1 displayed a common stem-loop structure at positions 11–27 nucleotides from the 3′ end, with five consecutive base pairs and a seven-base loop (Fig. 2C). The stem-loop region contained the majority of the commonly observed nucleotides, as represented in the most dominant sequence in N_20_-f1, named f1-1. Secondary structural prediction showed the same stem-loop structure at the same positions in 68% and 52% of ≥27 nt sequences in N_20_-f1 and N_20_>p-f1, respectively. In addition, only 7% of the RNAs in either family could form more than five base pairs in the stem region, underscoring the dominance of the specific stem-loop structure.

**Fig. 3:**
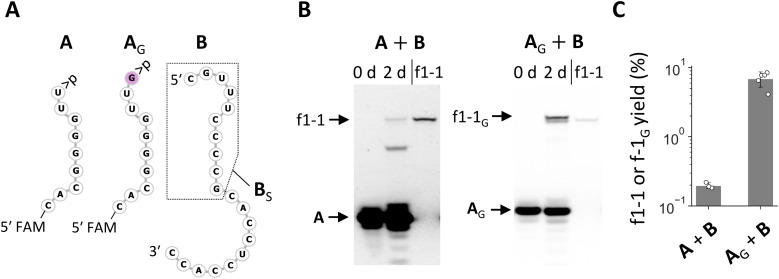
Synthetic pathways to the enriched RNA and its variant. (A) RNA sequences of fragments **A**, **A**_G_, and **B**. The 5′ ends of **A** and **A**_G_ were labeled with FAM for visualization. A portion of **B** enclosed by the dotted line corresponds to **B**_S_. (B) Incubation of **A** or **A**_G_ with **B** (20 μM each) in 100 mM MgCl_2_ at 22 °C for 2 days, analyzed by 20% denaturing PAGE. Pure f1-1 was run in parallel as a size control. (C) Yields of f1-1 and f1-1_G_ quantified from fluorescence intensities. Error bars indicate standard deviations (*n* ≥ 3).

The enrichment of RNA families with shared nucleotides and structures in the random RNA pools encouraged us to investigate how these sequences could have been synthesized. As they were observed in both N_20_ and N_20_>p pools, they should form *via* recombination. The conserved 3′ region in the RNA of varying lengths, in conjunction with the current understanding of recombination mechanisms, suggests a two-step α/α′ recombination, wherein hydrolysis forms >p at the 3′ end of one RNA, followed by ligation of the 5′-OH of another RNA to the >p.^31^ If the ligating RNA is 20 nt long, as in the original pools, the probable recombination junction was between the oft-observed C and U at positions 20 and 21 from the 3′ end. We first tested whether f1-1 (29 nt) can form through this mechanism by splitting f1-1 into the first 9 nt attached with >p (*i.e.*, fragment **A**) and the remaining 20 nt (*i.e.*, fragment **B**) (Fig. 3A) so they could undergo ligation, the second step of α/α′ recombination. In a 2 day incubation of **A** and **B**, we detected f1-1 with ∼0.2% yield (Fig. 3B and C). It is important to note that this reaction may not strictly reflect what happened in the original random RNA pools because other RNAs could have been involved.

We also tested recombination directly by attaching 11 nt random nucleotides to **A** (**A**_N_11__). Incubation of **A**_N_11__ with **B** did generate a distinguishable product whose length is similar to—but slightly longer than—f1-1 (Fig. S5A and S5B†). Sequence analysis of the product revealed that it was predominantly f1-1 with a G inserted between positions 20 and 21, named f1-1_G_ (Fig. S5C†). We confirmed that the addition of a G at the 3′ end of **A** (**A**_G_) (Fig. 3A) significantly enhanced its ligation with **B** (Fig. 3B and C). We also examined the effect of other nucleotides A, U, or C at the same position (**A**_A_, **A**_U_, or **A**_C_) for ligation with **B**. The fragment **A**_A_ exhibited improved ligation but less efficiently so than **A**_G_, whereas **A**_U_ and **A**_C_ did not show enhanced ligation (Fig. S6†). These variant RNAs were not detected in the products derived from the N_20_ and N_20_>p pools, despite only a single nucleotide difference from f1-1 and high capacity for synthesis, highlighting the difficulty of understanding reactions in random RNA mixtures based on an examination of only a small number of isolated RNAs.

### Discovery of a minimal self-reproducing RNA

**Fig. 4:**
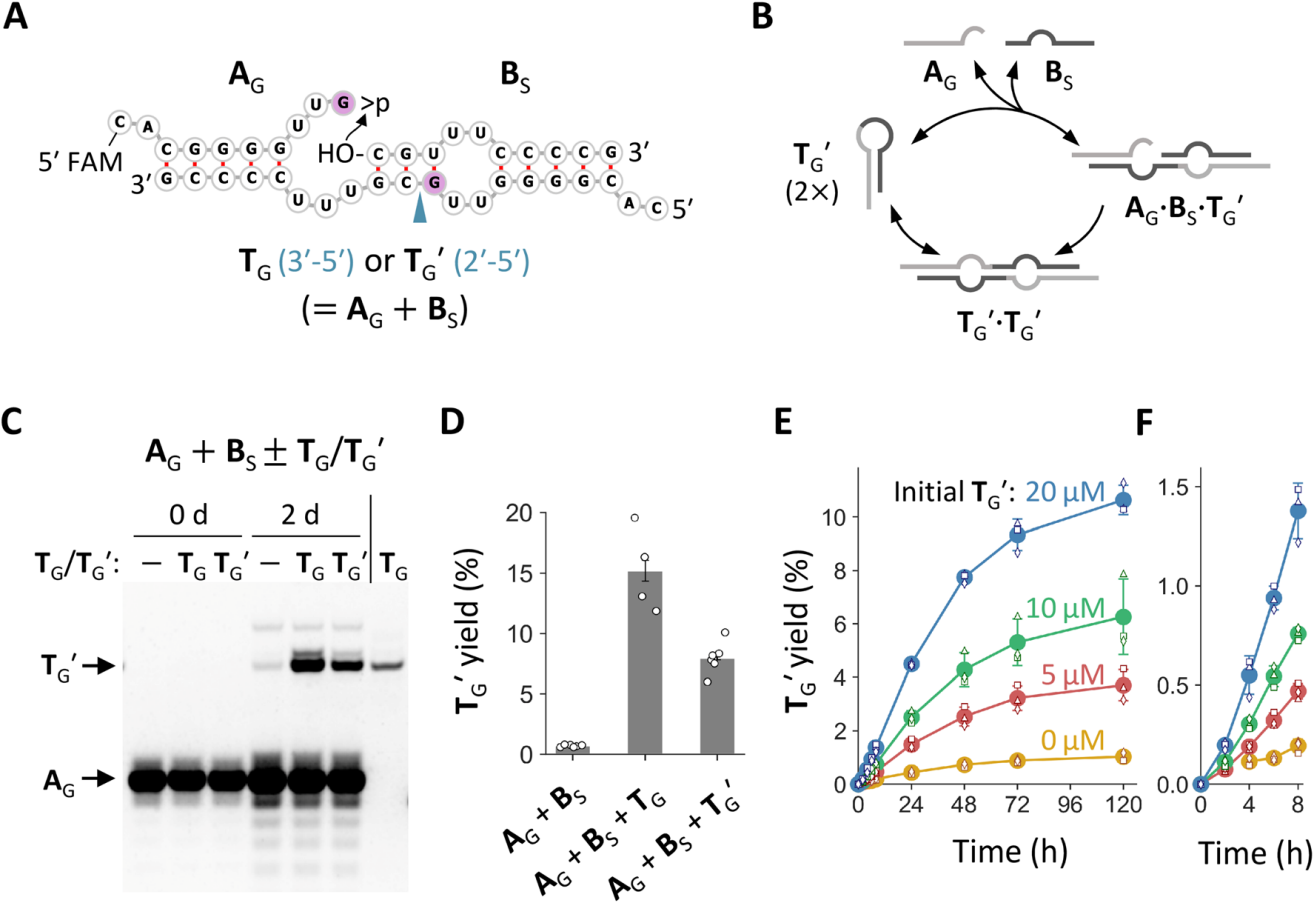
Minimal self-reproducing RNA. (A) Expected secondary structures of ternary complexes **T**_G_·**A**·**B**_S_ and **T**_G_′·**A**_G_·**B**_S_. The 5′ end of **A**_G_ was labeled with FAM for visualization. The G insertion is colored light purple. The arrowhead indicates a phosphodiester bond formed by ligation of **A** and **B**_S_, either a 3′–5′ or a 2′–5′ linkage. (B) Possible reproduction cycle of RNA (**T**_G_′ as an example). (C) Incubation of **A**_G_ and **B**_S_ (20 μM each) in the presence or absence of 20 μM **T**_G_ or **T**_G_′ in 100 mM MgCl_2_ at 22 °C for 2 days, analyzed by 20% denaturing PAGE. Pure **T**_G_ was run in parallel as a size control. (D) Yields of **T**_G_′ quantified from fluorescence intensities. Error bars indicate standard errors (*n* ≥ 3). (E) Time course of ligation between **A**_G_ and **B**_S_ (20 μM each) in the presence of 0–20 μM **T**_G_′. Filled circles represent the average yields of **T**_G_**T**_G_′ from three different trials (shown as different open symbols). Error bars indicate standard deviations. (F) An enlarged view of the plot in panel E for the first 8 h. Ligation in the absence of **T**_G_′ was undetected at 2 h.

We noticed that the common stem-loop structure in N_20_-f1 and N_20_>p-f1 (Fig. 2C) and their variants with the G insertion could catalyze the ligation between the 5′ and 3′ regions of themselves as a template, *i.e.*, self-reproduction (Fig. S7A,†4A, and B). In particular, nucleotide pairings around the ligation junctions upon ternary complex formation could enhance the ligation by positioning the termini of the two RNA substrates more proximally. We tested this hypothesis using the stem-loop regions of f1-1 and its variant with G at the ligation site, named **T** and **T**_G_, respectively (Fig. S7A† and 4A). We incubated 20 μM each of the 5′ regions with >p (**A** or **A**_G_) and the 3′ region (**B**_S_, the first 10 nt of **B**) for 2 days in the absence or presence of 20 μM **T** or **T**_G_. Whereas **T** improved ligation between **A** and **B**_S_ only slightly (∼1.4 fold) (Fig. S7B and S7C†), **T**_G_ enhanced ligation between **A**_G_ and **B**_S_ far more noticeably (∼21-fold) (Fig. 4C and D), demonstrating possible self-reproduction. We also tested the same reaction using **A**_A_, **A**_U_, and **A**_C_ and corresponding templates (**T**_A_, **T**_U_, and **T**_C_, respectively) instead of **A**_G_ and **T**_G_ (Fig. S8†). Although **T**_A_ and **T**_U_ catalyzed ligations between **A**_A_ or **A**_U_ and **B**_S_, their spontaneous ligations relative to the template-directed reactions were more productive than that of **A**_G_ and **B**_S_. The fragment **T**_C_ did not affect the ligation between **A**_C_ and **B**_S_.

Ligation between >p of **A**_G_ and **B**_S_ could generate two possible phosphodiester bonds, either 3′–5′ or 2′–5′ linkages (Fig. 4A). Using ribonuclease (RNase) T1, which selectively cleaves G3′-p-5′N linkages of unpaired nucleotides, we determined that the ligation catalyzed by **T**_G_ primarily formed a 2′–5′ linkage (Fig. S9†). Next, we prepared **T**_G_ containing a 2′–5′ linkage at the ligation junction and named it **T**_G_′. We confirmed that **T**_G_′ catalyzed the same ligation reaction to generate more of itself (Fig. 4C and S9†), demonstrating true self-reproduction (Fig. 4B), although the extent of catalysis was approximately half than that of **T**_G_ (Fig. 4D). Whereas previous studies found that RNA containing a fraction of 2′–5′ linkages can assist non-enzymatic RNA polymerization^39^ and retain functions as aptamers or ribozymes,^40^ our study further showed that such RNA can also self-reproduce. A time course experiment revealed the gradual appearance of **T**_G_′, with the reaction slowing after a 2 day (48 h) incubation (Fig. 4E, F and S10†). The yield of **T**_G_′ was positively increased with the concentration of initial **T**_G_′, demonstrating its autocatalytic ability. The ligation between >p of **A**_G_ and **B**_S_ was confirmed by control reactions performed in the absence of >p or **B**_S_, which showed negligible **T**_G_′ reproduction (Fig. S11†). We also found that the self-reproduction of **T**_G_′ was substantially enhanced at high concentration of Mg^2+^ (100 mM MgCl_2_) and temperatures around 22 °C (Fig. S12†), the condition used for incubating the original random RNA pools (Fig. 1A).

**Fig. 5:**
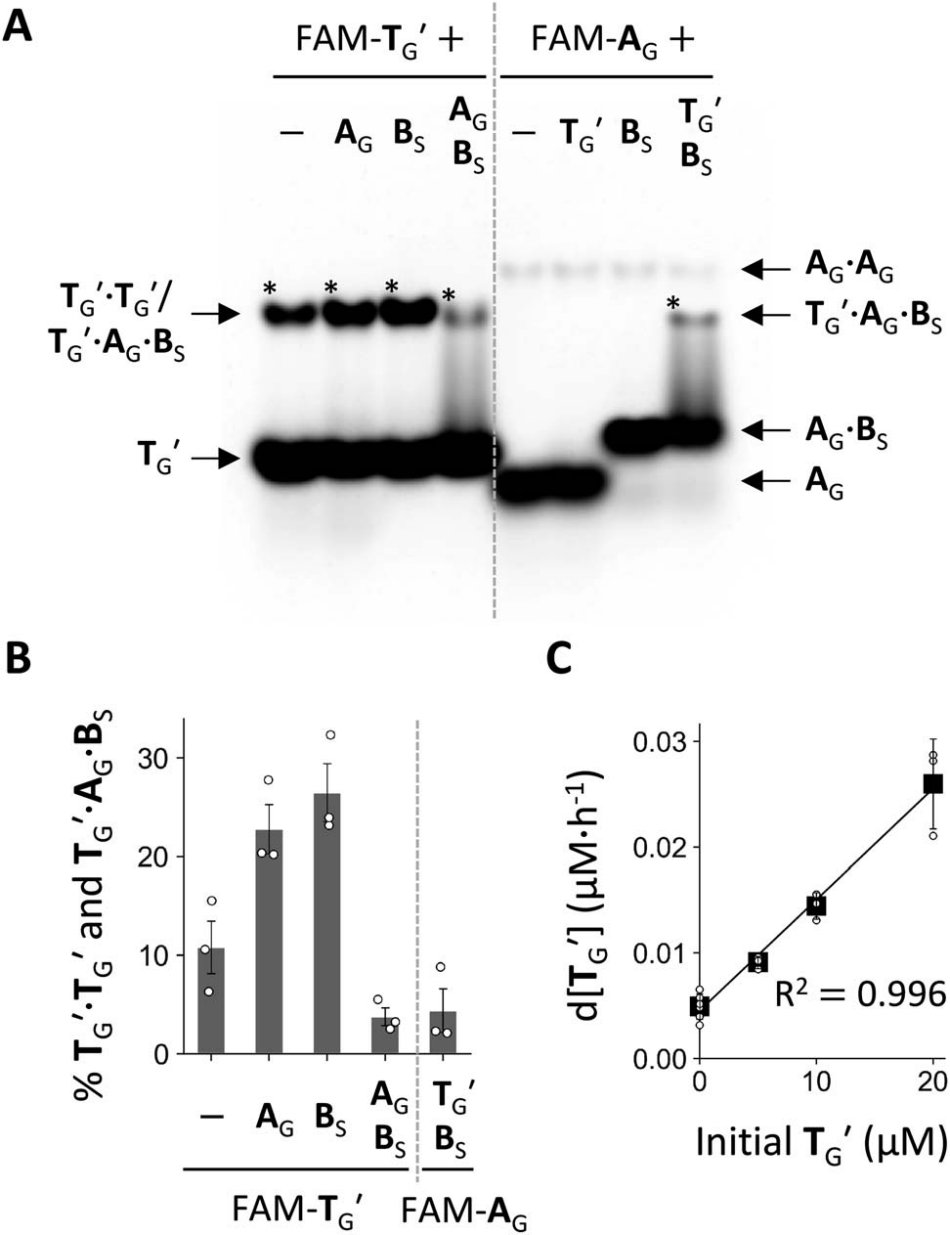
Characteristics of the self-reproducing RNA. (A) Native PAGE analysis of RNA mixtures. Various combinations of **A**_G_, **B**_S_, and **T**_G_′ (20 μM each) containing FAM-labeled **T**_G_′ or **A**_G_ were incubated in 100 mM MgCl_2_ at 22 °C for 6 h, and then immediately subjected to 20% native PAGE in 20 mM MgCl_2_ at 22 °C. Asterisks indicate complexes whose percentages were quantified in panel B. (B) Percentages of **T**_G_′·**T**_G_′ and **T**_G_′·**A**_G_·**B**_S_ complexes calculated as the ratio of the fluorescence intensities of the bands to summed intensities of all observed bands. Error bars indicate standard errors (*n* = 3). (C) Initial rate of **T**_G_′·**T**_G_′ formation as a function of initial concentration (0–20 μM) of **T**_G_′·**T**_G_′ for ligation between **A**_G_ and **B**_S_ (20 μM each) in 100 mM MgCl_2_ at 22 °C. Black squares show average rates from different experiments (represented as dots) fitted to the autocatalytic equation with *p* = 1 (black line). Error bars indicated standard deviations (*n* ≥ 3).

Next, we examined the formation of higher-order complexes among **A**_G_, **B**_S_, and **T**_G_′ by native PAGE after co-incubating one, two, or three of these RNAs containing fluorescently labeled **T**_G_′ (FAM-**T**_G_) or **A**_G_ (FAM-**A**_G_) for 6 h (Fig. 5A). In this experiment, **A**_G_ contained a monophosphate (-p) instead of >p at the 3′ end to preclude ligation to **B**_S_ (Fig. S11†). When incubating only **T**_G_′, we found that the majority of **T**_G_′ existed as a **T**_G_′ monomer, with only a fraction (∼11%) forming a **T**_G_′·**T**_G_′ dimer (Fig. 5B). A **T**_G_′·**T**_G_′ dimer is presumably a simple self-complementary template dimer (Fig. S14†), but two **T**_G_′ molecules may also interact by forming a kissing loop. The prevention of the formation of a **T**_G_′·**T**_G_′ dimer was partly due to the 2′–5′ linkage, which significantly reduced the dimerization of **T**_G_′ (Fig. S13†), consistent with previous studies showing the diminished thermal stability of RNA duplexes in the presence of 2′–5′ linkages.^40,41^ The amount of **T**_G_′·**T**_G_′ increased to 23–27% in the presence of either **A**_G_ or **B**_S_. However, in the presence of both **A**_G_ and **B**_S_, the total amount of the **T**_G_′·**T**_G_′ dimer and a **T**_G_′·**A**_G_·**B**_S_ ternary complex decreased to ∼3.8%. When incubating the three RNA molecules with FAM-**A**_G_, we detected the formation of a comparable amount of the **T**_G_′·**A**_G_·**B**_S_ complex. In addition, we found that the majority (∼80%) of **A**_G_ was bound to **B**_S_, and thus most of the substrates were not freely available, which could explain the low percentage of the **T**_G_′·**A**_G_·**B**_S_ complex formation and the limited self-reproduction of **T**_G_ (Fig. 4E).

The high availability of **T**_G_′ as a monomer implies its potential to undergo non-linear amplification by circumventing the strong association of two self-complementary **T**_G_′ molecules that form after ligation of **A**_G_ and **B**_S_ (Fig. 4B). A common way of examining such a possibility for a template (or an autocatalyst) is to fit the initial rate of its own production to the model of self-reproduction:^13,14,23,24,42^
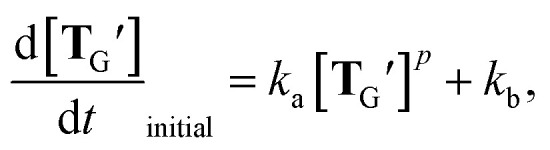
where *k*_a_, *k*_b_, and *p* represent the autocatalytic rate enhancement, the background reaction rate, and the reaction order, respectively. We doped varied concentrations of **T**_G_′ into a mixture of fixed concentrations of **A**_G_ and **B**_S_ and investigated the enhancement of the initial reaction rate (Fig. 4F and 5C). The concentrations of **T**_G_′ were chosen so that the fraction of the **T**_G_′·**A**_G_·**B**_S_ complex was sufficiently small compared with the total amount of substrates^42^ (*cf.*Fig. 5B), as in a previous study.^13^ As expected, the initial rate of **T**_G_′ formation increased with the initial concentrations of **T**_G_′. Furthermore, the initial rates can be fit well (*R*^2^ = 0.996) with the self-reproduction equation by assuming *p* = 1, corresponding to exponential growth. This result indicates the potential of **T**_G_′ to undergo exponential self-reproduction. We estimated *k*_a_ and *k*_b_ as 0.0011 ± 0.000069 h^−1^ and *k*_b_ = (0.0045 ± 0.00070) × 10^−6^ M h^−1^. The autocatalytic efficiency (*k*_a_/*k*_b_)^42^ of **T**_G_′ (2.4 × 10^5^) is comparable to or lower than a much larger recombination or ligase ribozyme,^13,14^ while higher than DNA-based self-reproduction systems^23–25^ with the caveat that they have smaller reaction orders (*p* = ∼0.5).

The RNA molecule **T**_G_′ shares many similarities with the previously engineered 61 nt self-reproducing ligase ribozyme,^13^ although they catalyzed different ligation chemistries (Fig. S14†). The ribozyme catalyzes the attack of the 3′-OH of an RNA substrate on a 5′ triphosphate of another substrate in a template-directed manner and generates a ligated product identical to the ribozyme. Its self-reproduction was limited because of the strong association of the two substrates, as is also observed in **T**_G_′ (Fig. 5A). Nevertheless, both systems exhibited high apparent autocatalytic reaction order (∼1) in an isothermal environment as a consequence of the weak self-binding of the templates, compared to other nucleotide-based template-directed self-reproduction systems that showed an order of ∼0.5.^23–25^ This could be partly attributed to the intramolecular structural formation of a template, G:U wobble pairs that can facilitate template-directed ligation while supporting dissociation of a duplex,^43^ and multiple thermodynamically unfavorable bulges in a dimer,^44^ all of which are commonly observed in both **T**_G_′ and the ligase ribozyme (Fig. S14†).

The limited self-reproduction of **T**_G_′ resulted from multiple factors. First, the 2′–5′ linkage, while reducing the dimerization of **T**_G_′, decreased the ligation efficiency (Fig. 4D). Second, **T**_G_′ did not efficiently form an active complex with the substrates **A**_G_ and **B**_S_ because most of the two substrates bound to each other and were not freely available (Fig. 5A). These limitations may be overcome if strong chemical activation is adopted instead of >p or in environments that periodically experience low pH, high temperatures, or low MgCl_2_ concentrations, which destabilize RNA–RNA interactions (*e.g.*, the association of substrates).^45–47^ Alternatively, as demonstrated for a self-reproducing ligase ribozyme,^48^ directed evolution with **T**_G_′ as the parent RNA may also identify highly efficient reproduction of oligonucleotides in a constant environment. It was shown that only a slight difference, including two critical mutations, was sufficient to convert the original ligase ribozyme^13^ (Fig. S14†) into a continuously self-reproducible RNA.^49^ Thus, it is conceivable that there may be a short RNA oligonucleotide capable of unlimited self-reproduction, in a sequence space accessible from **T**_G_′ by natural selection.

## Conclusions

We demonstrated a form of minimal RNA self-reproduction driven by prebiotically plausible chemistry, providing a potential missing link between abiotic oligomers and the eventual emergence of a genetic system. The 20 nt RNA, **T**_G_′, accelerated >p-dependent ligation between two 10 nt substrates, **A**_G_ and **B**_S_, as a template for generating identical **T**_G_′ molecules (Fig. 4C and S9†). Such self-reproduction of RNA could have occurred in the RNA World because RNA of these lengths can be generated non-enzymatically,^18,19^ and >p can also be readily formed by spontaneous RNA hydrolysis or with prebiotically plausible reagents.^32,33^ Although >p is eventually hydrolyzed to monophosphates, *in situ* reactivation back to >p^33^ could extend the self-reproduction of **T**_G_′, which is currently limited (Fig. 4E). The self-reproduction was also supported by a 2′–5′ phosphodiester bond, which is thought to have been prevalent in primordial RNA pools as generated in typical non-enzymatic RNA synthesis.^36–38^ Short RNA molecules capable of self-reproduction by template-directed ligation, as shown in the present study, has been proposed as the earliest stage toward the evolution of complex replication ribozymes.^21,22^ Our results complement this view and help delineate the development of RNA-based genetic systems during the origins of life.

Our results also give insights into the dynamics of short random RNA mixtures. From completely random pool of 20-mers, we identified a discrete class of related, enriched sequences of which f1-1 appeared to be a canonical representative. The fragment **T**_G_′ is a truncated version of f1-1_G_, a single-mutation variant of f1-1. Both f1-1_G_ and f1-1 were accessible products in both N_20_ and N_20_>p pools explored in the present study. However, while f1-1 was highly enriched in both random RNA pools along with many related sequences (*e.g.*, N_20_-f1 and N_20_>p-f1), f1-1_G_ was undetected even at a low frequency. On the other hand, biochemical analyses revealed the superiority of f1-1_G_ to f1-1 for its formation through simple ligation of two substrate fragments (Fig. 3B and C). This discrepancy may imply the involvement of other RNA species for the synthesis of f1-1 in the random RNA pools. In the chaos of primordial soup, it is without question that a complex ecology of chemical reactions must have given rise to enriched species sets.^50,51^ A previous study also reported the inefficient synthesis of some products isolated from random RNA pools.^35^ Altogether, our results highlight the difficulty of inferring dominant reactions in random RNA mixtures from the analyses of isolated sequences. Nevertheless, the information obtained from examining the random RNA products was valuable in the discovery of the minimal self-reproducing RNA, which exhibited its highest activity in the original environment where the random RNA pools were exposed (Fig. S12†). Future experiments exploring the synthesis of f1-1, f1-1_G_, or **T**_G_′ in combination with random RNA mixtures would give more insights into the likelihood of the emergence of self-reproduction in a primordial RNA soup.

## Data availability

The data supporting the findings of this study are available from the corresponding author upon reasonable request.

## Author contributions

R. M. and N. I. designed the project. R. M. performed experiments, analyzed data, and wrote the paper with comments from N. I.

## Conflicts of interest

There are no conflicts to declare.

## Supplementary Material

SC-014-D3SC01940C-s001
