# Supplementary material for: Minimal RNA self-reproduction discovered from a random pool of oligomers
Source: Chem Sci. 2023 Jun 20;14(28):7656–64. doi: 10.1039/d3sc01940c (PMC10355099; doi:10.1039/d3sc01940c)
Supplement: SC-014-D3SC01940C-s001 [file SC-014-D3SC01940C-s001.pdf]

Electronic Supplementary Material (ESI) for Chemical Science.
This journal is © The Royal Society of Chemistry 2023

Supplementary Information for

Minimal RNA self-reproduction discovered from a random pool of oligomers

Ryo Mizuuchi*, Norikazu Ichihashi

*Email: mizuuchi@waseda.jp

This file includes:

Materials and Methods
Figs. S1 to S14
Tables S1
References

Materials and Methods

RNA preparation

All RNAs, except those containing a 2′-5′ linkage or terminal >p, were purchased from Integrated DNA Technologies (IDT, Coralville, IA). The RNAs containing a 2′-5′ linkage were purchased from Hokkaido System Science Co., Ltd. (Hokkaido, Japan). RNAs with >p were generated by treating RNAs with a terminal monophosphate with 20 mM 1-(3-dimethylaminopropyl)-3-ethylcarbodiimide hydrochloride (EDC·HCl, Nakaraitesk) in 300 mM MES buffer (pH 5.5) for 30 min at 37 °C, according to a previous study.[1] The >p-activated RNAs, except for N20>p, were purified by 20% polyacrylamide / 8 M urea denaturing PAGE in 1×TBE buffer. The RNAs were ethanol precipitated and rehydrated in 0.1 mM EDTA (pH 8.0). The RNA sequences are listed in Table S1.

Denaturing PAGE

An aliquot of RNA mixture was added to four volumes of a quenching buffer (90% formamide, 50 mM EDTA, pH 8.0, 0.025% bromophenol blue). Samples were then heated to 80 ºC for 2 min, cooled on ice for 1 min, loaded onto a 20% polyacrylamide / 8 M urea denaturing gel (14 cm × 7 cm × 1 mm) in 1×TBE buffer, and electrophoresed at 270 V for approximately 100 min. FAM-labeled RNAs were visualized directly, while unlabeled RNAs were imaged after staining with SYBR Gold, using FUSION-SL4 (Vilber-Lourmat). The obtained images were analyzed using ImageJ (NIH), and ligation yields were calculated as the ratio of the fluorescence intensity of RNA products to that of the original FAM-labeled RNA substrates at time 0.

Collection of reaction products from random RNA pools

Random RNA oligomers at a concentration of 50 μM (N_{20} or N_{20}>p) were incubated in 50 mM Tris-HCl (pH 8.0) and 100 mM $MgCl_2$ at 22 °C for 2 d. The RNAs were heated to 65 ºC for 2 min and cooled to room temperature before use. Following incubation, RNAs (~100 pmol) derived from N_{20}>p were ethanol precipitated and treated with 10 U T4 PNK (NEB) at 37 °C for 1 h to remove the >p moiety. The RNA samples were then subjected to denaturing PAGE, as described above, and RNA products were excised from a denaturing gel and ethanol precipitated. Subsequently, the RNA products were polyadenylated, reverse transcribed, and PCR-amplified using the SMARTer smRNA-Seq Kit for Illumina (Takara), according to the manufacturer's protocol. The Custom Read2 Primer described in the protocol was used for reverse transcription, and primers from the SMARTer RNA Unique Dual Index Kit (Takara) were used for PCR. The cDNA samples were purified on a 15% native polyacrylamide gel (SuperSep DNA, Fujifilm)

and sequenced using DNBSEQ-G400 (PE100) at BGI Hong Kong. The nucleotide sequence compositions of the original N_{20} and N_{20}>p pools were also determined using the same method.

Nucleotide sequence analysis

Raw sequence reads were trimmed following the manufacturer's protocol of the SMARTer smRNA-Seq Kit for Illumina (Takara) by using cutadapt.[2] The reads were further quality-filtered (>Q20), and primer-derived reads were removed. All 21–45-nt reads counted at least twice were analyzed for products derived from the N_{20} and N_{20}>p pools, while all 20 nt reads were analyzed for the original pools. The base compositions of products of each length (Figure 1D) were visualized by converting the frequency of each nucleotide (f_N, N = A, U, G, or C) at each position into RGB codes (R, G, B) according to a previous study,[3] using the following equations:

$$R = 255(1 - f_C - f_A),$$

$$G = 255(1 - f_C - f_U),$$

$$B = 255(1 - f_A - f_G - f_U),$$

and

$$\sum f_N = 1.$$

RNA secondary structures were predicted as minimal free energy structures at 22 °C using the Vienna RNA Package.[4] The most abundant 10,000 sequences derived from N_{20} or N_{20}>p pools were assigned to families such that 21 nt of 5′ or 3′ terminus of each sequence differs by ≤7 edits from the most abundant product in the same family. Sequences in each family were aligned using the MAFFT online service with default settings,[5] and the probabilities of bases at each position were visualized using the python package Logomaker.[6] RNA secondary structures were displayed using Forna.[7]

RNA-RNA ligation/recombination reactions

RNAs of choice were incubated in 50 mM Tris-HCl (pH 8.0) and 100 mM MgCl2 at 22 °C. The RNAs were heated to 65 °C for 2 min and cooled to room temperature before use. The RNA concentrations and incubation times were specified in the text or figure captions. After incubation, RNA samples were analyzed by denaturing PAGE, as described above. The initial rate of $\mathbf{T}_G'$ synthesis was estimated by a linear fit to data points up to 4 h, at which the extent of reaction was 4.9–11% of a maximum yield at 5 d. The rates were then fitted to the model of self-reproduction using the Python package

scipy.optimize.curve_fit to obtain associated parameters. The recombination products between $\mathbf{A}_{N11}$ and **B** were excised from a denaturing gel after SYBR Gold staining, ethanol precipitated, polyadenylated, and reverse transcribed using primer 1 (Table S1), as described above. The products were PCR-amplified using primers 2 and 3 (Table S1), followed by purification on a 15% native polyacrylamide gel. Then, the products were amplified by another PCR using primers 3 and 4 (Table S1), purified in the same method, and subjected to Sanger nucleotide sequence analysis.

RNAse T1 treatment

Each RNA (~ 4 ng) was incubated with or without 0.2 U RNAse T1 in 50 mM Tris-HCl (pH 7.6) and 30 mM $MgCl_2$ at 22 °C for 1 h. An aliquot of the reaction mixture was added to two volumes of the quenching buffer and analyzed by denaturing PAGE as described above.

Native PAGE

One, two, or three RNAs of choice (20 μM each) were incubated in 50 mM Tris-HCl (pH 8.0) and 100 mM $MgCl_2$ at 22 °C for 6 h. The fragment $\mathbf{A}_G$ was attached with a 3′ monophosphate instead of >p to preclude ligation. An aliquot of the RNA samples was mixed with a 2×native-gel loading dye (50 mM Tris-HCl (pH 8.0), 100 mM $MgCl_2$, 0.1% bromophenol blue, and 20% v/v glycerol) and immediately loaded onto a 20% polyacrylamide gel containing 20 mM $MgCl_2$. The gel was electrophoresed at 22 °C and 40 V for approximately 14 h, and FAM-labeled RNAs were visualized using FUSION-SL4 (Vilber-Lourmat).

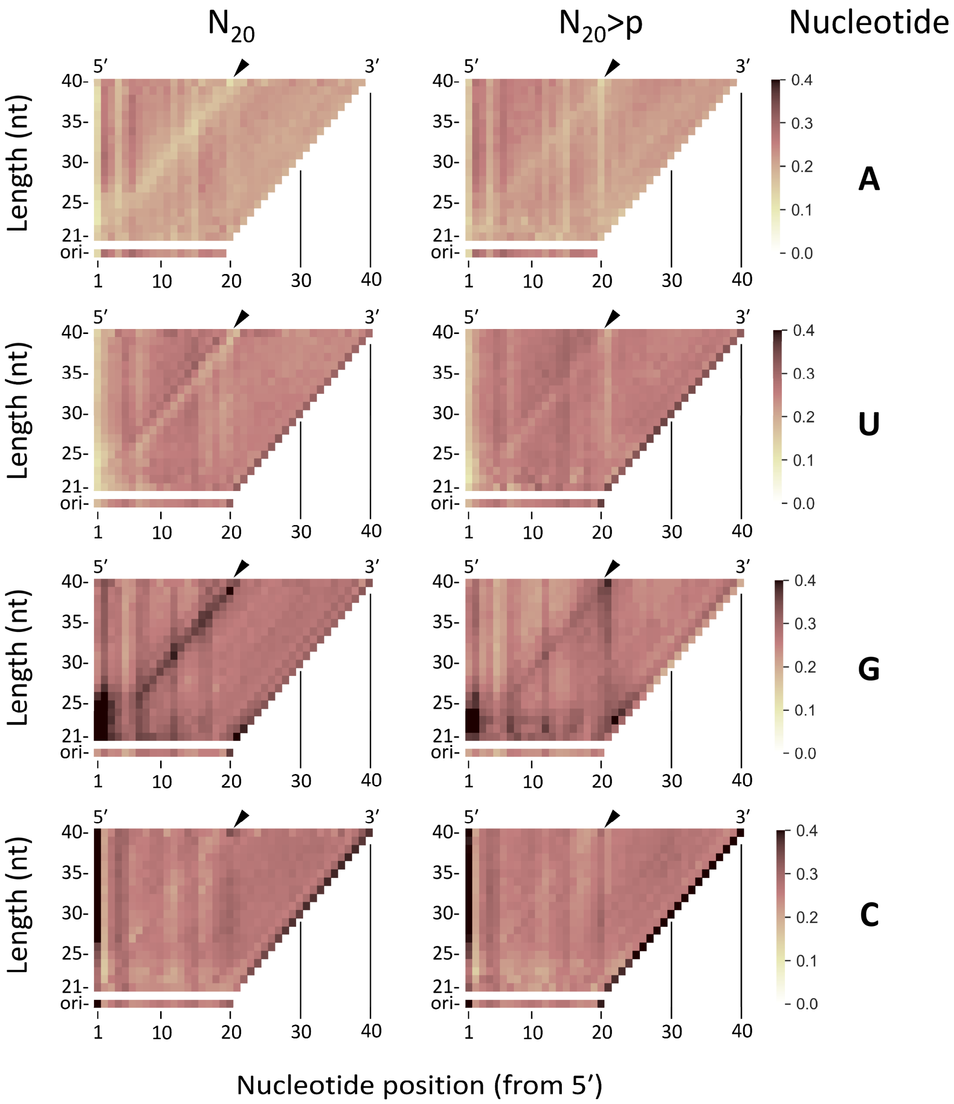

Fig. S1. Nucleotide compositions of the RNA products (21–40 nt) with each length for N20 (left) and N20>p (right) pools. Each heatmap shows the frequency distribution of one type of nucleotide. The distributions of the original 20 nt pools were displayed for comparison. Arrowheads indicate the putative ligation junctions.

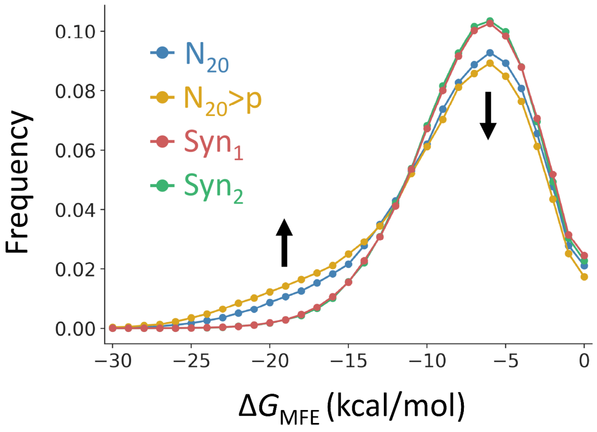

Fig. S2. Predicted free energies of minimal free energy structures (ΔG_{MFE}) of RNA products derived from N_{20} and N_{20}>p. The same products investigated in Fig. 1 were analyzed. "Syn_1" and "Syn_2" represent synthetic sequences generated based on the length distributions (Fig. 1C) and nucleotide compositions (Fig. 1D) for N_{20} and N_{20}>p, respectively. Arrows indicate clear free energy changes between experimental and synthetic sequences.

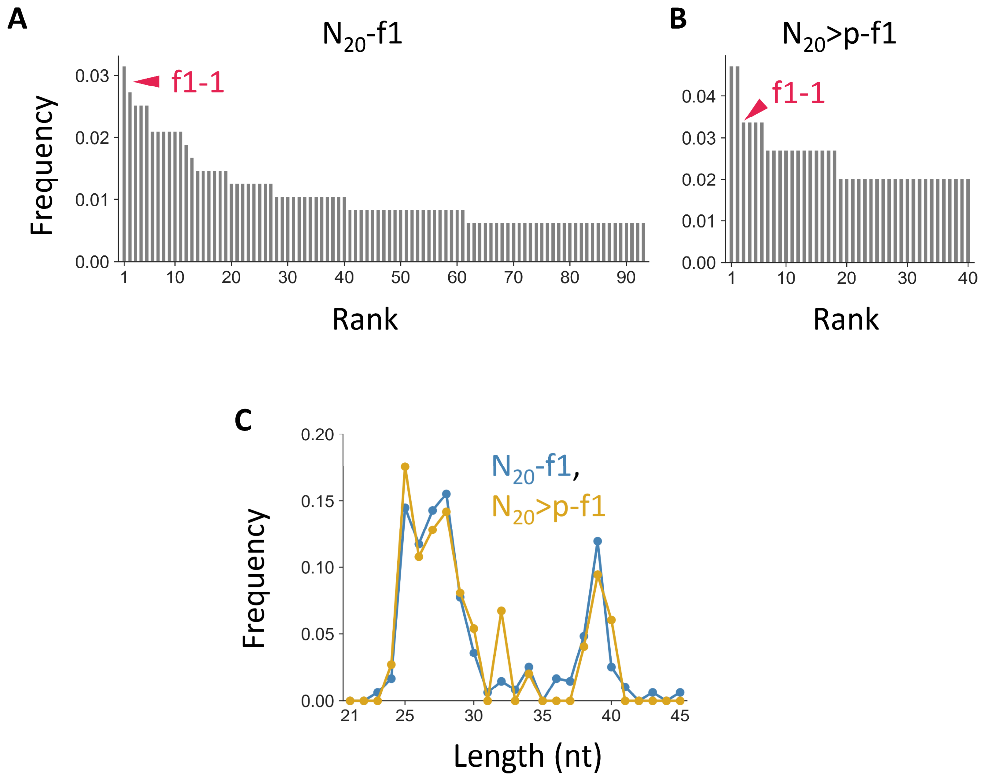

Fig. S3 Abundances of sequences in the most enriched families. (A, B) Frequencies of all sequences in (A) N_{20}-f1 and (B) N_{20}>p-f1 in total reads of each family, sorted in descending order. Magenta arrowheads indicate f1-1. (C) Length distributions of sequences in each family.

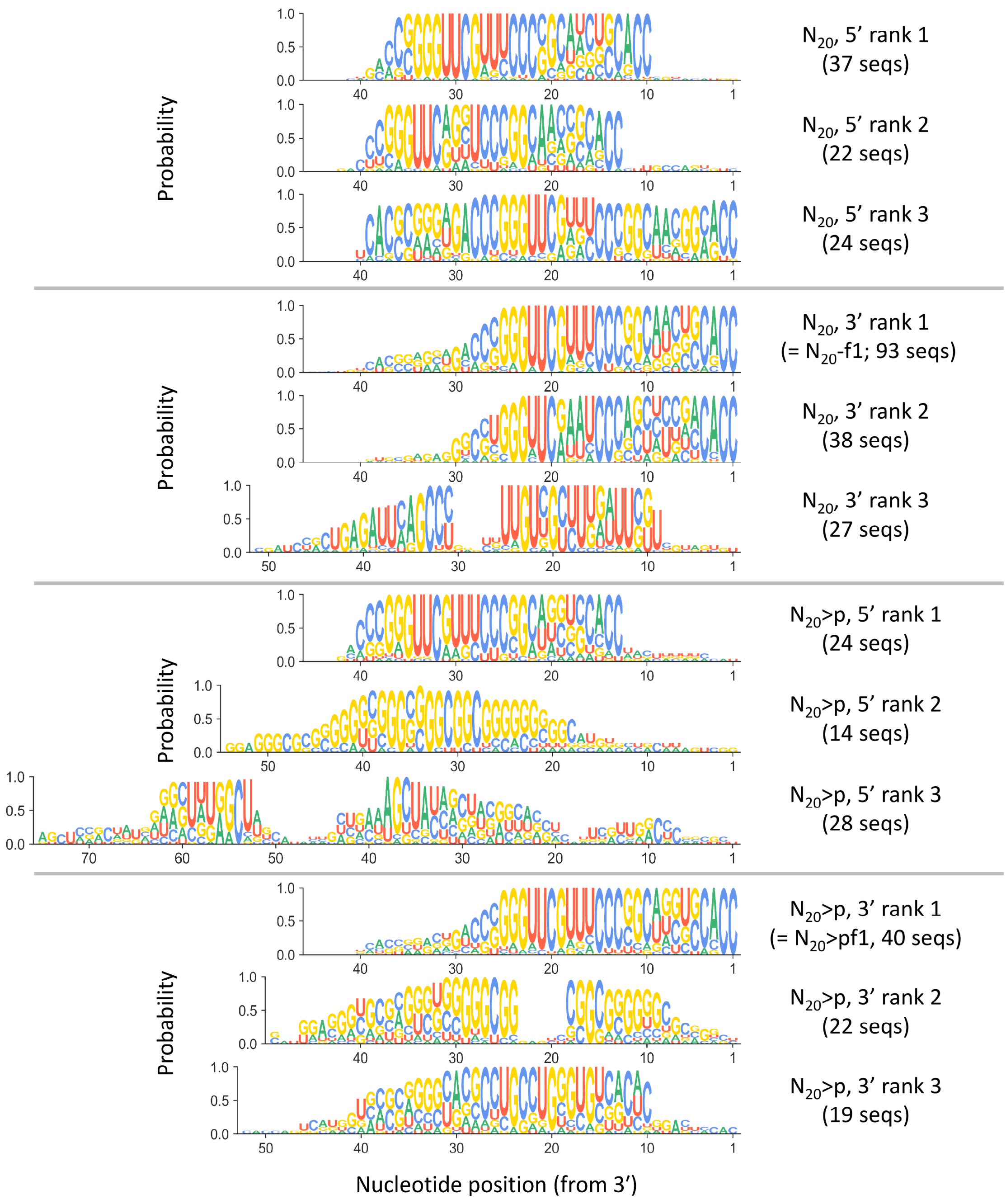

Fig. S4. Nucleotide conservation values in the most enriched RNA families. The three most enriched families, when grouped based on sequence similarity around 5′ or 3′ terminus for N_{20} or N_{20}>p, were analyzed as in Fig. 2B. The number of sequences ("seqs") for each family is shown.

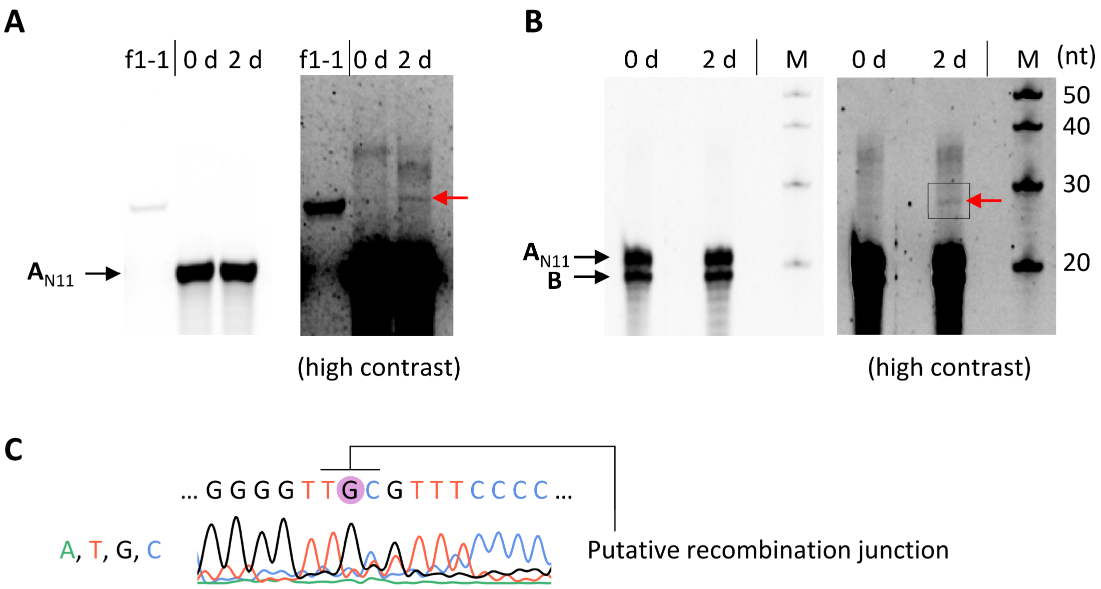

Fig. S5. Recombination between $\mathbf{A}_{N11}$ and **B**. (A) Incubation of FAM-labeled $\mathbf{A}_{N11}$ and **B** (20 µM each) in 100 mM $MgCl_2$ at 22 °C for 2 d, analyzed by 20% denaturing PAGE. Pure f1-1 was run in parallel as a size control. The red arrow indicates products of interest. (B) Unlabeled $\mathbf{A}_{N11}$ and **B** (40 µM each) were incubated in the same condition and analyzed by 20% denaturing PAGE and SYBR Gold staining. The black rectangle indicates the region excised for RT-PCR and sequencing. (C) Nucleotide sequence chromatogram of a part of the RT-PCR products. The dominant sequence was shown above, with the light purple circle indicating the insertion of G at the putative recombination junction.

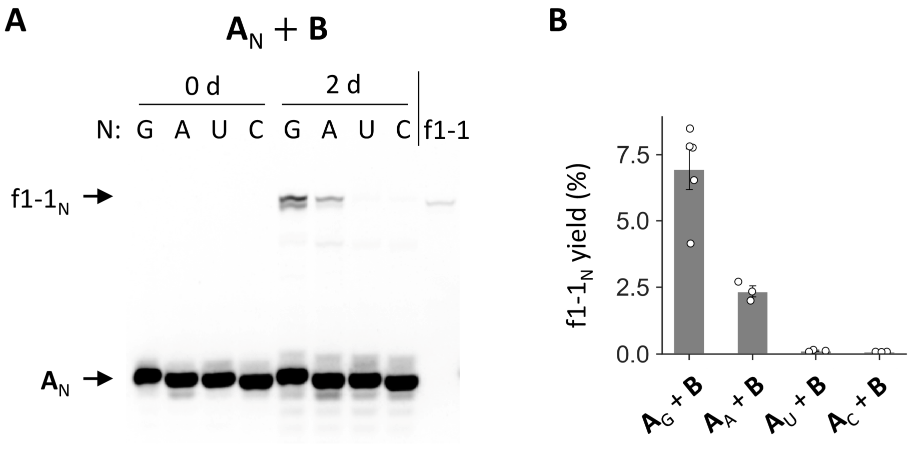

Fig. S6. Ligation between the variants of $\mathbf{A}_G$ and **B**. (A) Incubation of FAM-labeled $\mathbf{A}_N$ (N = G, A, U or C) and **B** (20 μM each) in 100 mM $MgCl_2$ at 22 °C for 2 d and analyzed by 20% denaturing PAGE. Pure f1-1 was run in parallel as a size control. Fragments $\mathbf{A}_N$ and f1-1_N represent sequences with a single nucleotide (N) addition at the 3′ end of **A** and ligation products between $\mathbf{A}_N$ and **B**, respectively. (B) Yields of f1-1_N quantified from fluorescence intensities. Data for $\mathbf{A}_G$ + **B** are the same as in Fig. 3C. Error bars indicate standard errors ($n \geq 3$).

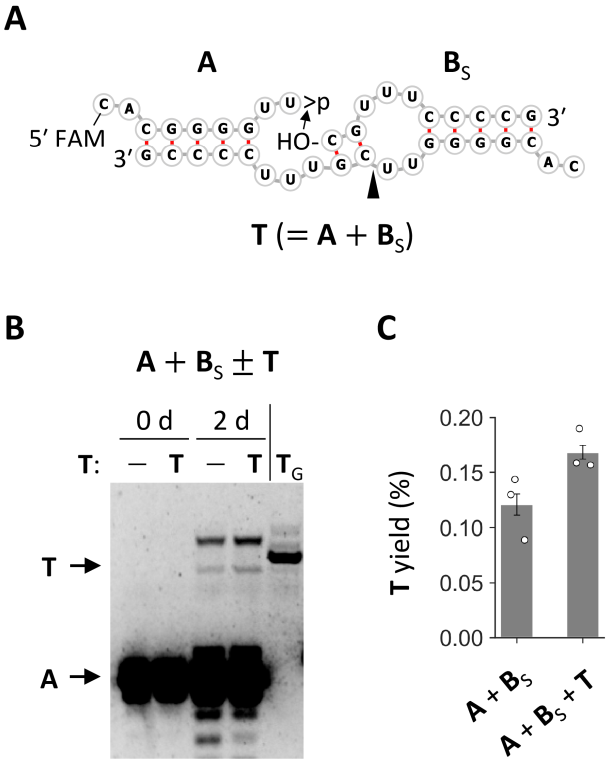

Fig. S7. Possible reproduction of **T**. (A) Expected secondary structures of ternary complexes **T**·**A**·**B**S. The 5′ end of **A** was labeled with FAM for visualization. The arrowhead indicates the phosphodiester linkage that forms by the ligation between **A** and **B**S. The chemical nature of the linkage (3′-5′ or 2′-5′) was not determined. (B) Incubation of **A** and **B**S (20 µM each) in the presence or absence of 20 µM **T** in 100 mM $MgCl_2$ at 22 °C for 2 d, analyzed by 20% denaturing PAGE. Pure **T**G was run in parallel as a size control. (C) Yields of **T** quantified from fluorescence intensities. Error bars indicate standard errors (n = 3).

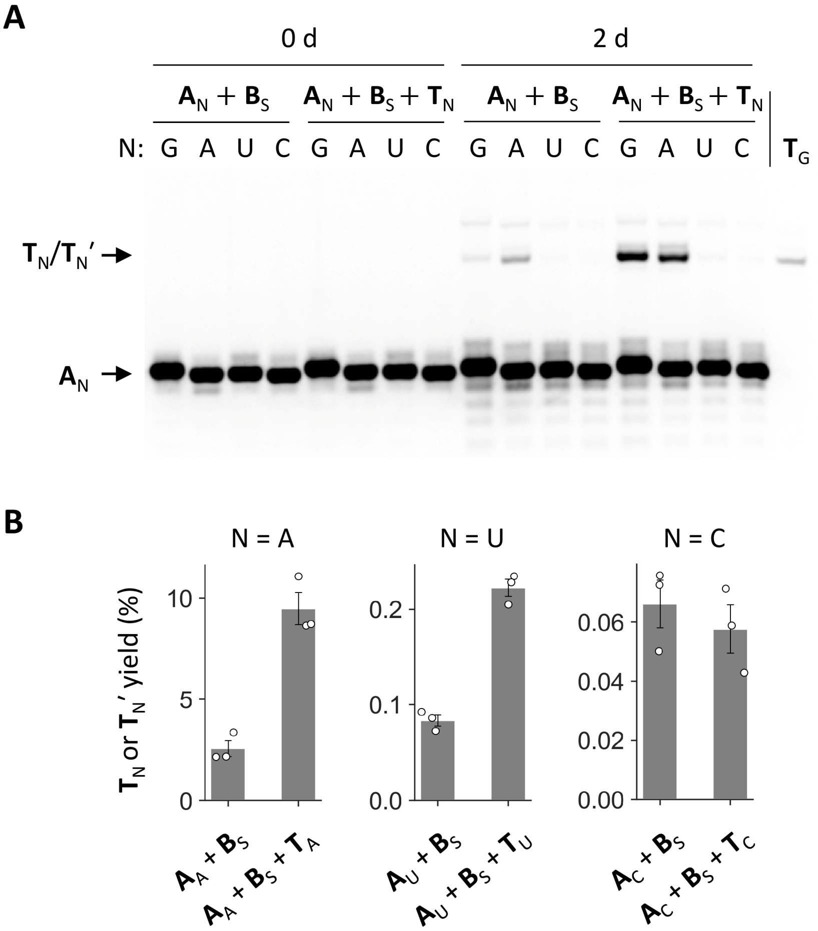

Fig. S8. Ligation between the variants of $\mathbf{A}_\mathrm{G}$ and $\mathbf{B}_\mathrm{S}$. (A) Incubation of FAM-labeled $\mathbf{A}_\mathrm{N}$ (N = G, A, U or C) and $\mathbf{B}_\mathrm{S}$ (20 μM each) in the presence or absence of 20 μM $\mathbf{T}_\mathrm{N}$ in 100 mM $MgCl_2$ at 22 °C for 2 d and analyzed by 20% denaturing PAGE. Pure $\mathbf{T}_\mathrm{G}$ was run in parallel as a size control. The symbols $\mathbf{A}_\mathrm{N}$ and $\mathbf{T}_\mathrm{N}$ denote sequences with a single nucleotide (N) addition at the 3′ end of **A** and ligation products between $\mathbf{A}_\mathrm{N}$ and $\mathbf{B}_\mathrm{S}$. (B) Yields of $\mathbf{T}_\mathrm{N}$ or $\mathbf{T}_\mathrm{N}$′ ($\mathbf{T}_\mathrm{N}$ with a 2′-5′ phosphodiester linkage at the ligation junction) for N = A, U, or C, quantified from fluorescence intensities. Error bars indicate standard errors (n = 3).

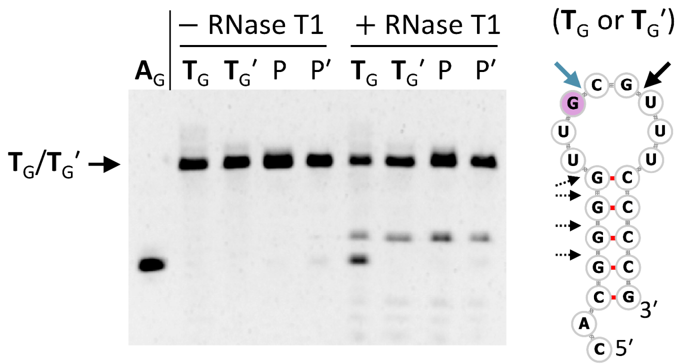

Fig. S9. Determination of a phosphodiester linkage formed upon ligation between $\mathbf{A}_{G}$ and $\mathbf{B}_{S}$. Incubation of $\mathbf{T}_{G}$, $\mathbf{T}_{G}'$, and products of ligation catalyzed by $\mathbf{T}_{G}$ (P) or $\mathbf{T}_{G}'$ (P′) in the presence or absence of RNase T1, analyzed by denaturing PAGE. All RNAs were labeled with 5′-FAM. Pure $\mathbf{A}_{G}$ was run in parallel as a size control. The predicted secondary structure of $\mathbf{T}_{G}$ (or $\mathbf{T}_{G}'$) is shown on the right. The thick arrows indicate the two major cleavable G3′-p-5′N linkages, whereas the dotted arrows indicate other G3′-p-5′N linkages. The blue arrow indicates the linkage of interest.

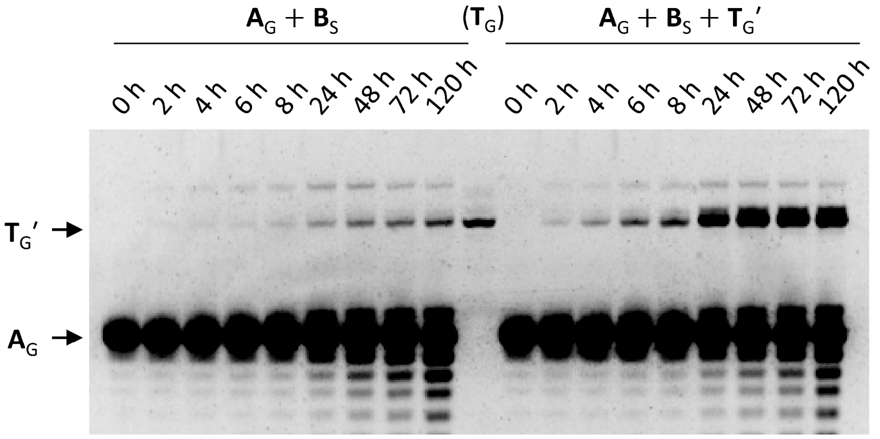

Fig. S10. An example of a denaturing PAGE gel from the time course experiment with **A**G, **B**S, and **T**G′. Pure **T**G was run in parallel as a size control.

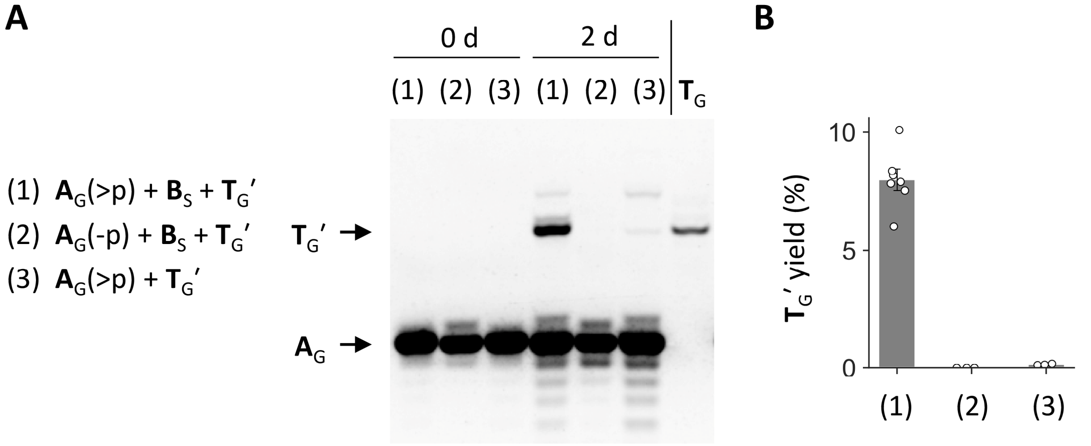

Fig. S11. Control experiments for the self-reproduction of **T**G′. (A) Incubation of FAM-labeled **A**G (with >p or -p at the 3′ end) and **T**G′ (20 µM each) in the presence or absence of 20 µM **B**S in 100 mM $MgCl_2$ at 22 °C for 2 d, analyzed by 20% denaturing PAGE. The combination of the RNAs used for each reaction is described in the panel. Pure **T**G was run in parallel as a size control. Note that, albeit inefficiently, the same ligation seemed to have occurred without **B**S (3), because **B**S can be generated by hydrolysis of **T**G′. (B) Yields of **T**G′ quantified from fluorescence intensities. Data for (1) are the same as in Fig. 4D. Error bars indicate standard errors ($n \geq 3$).

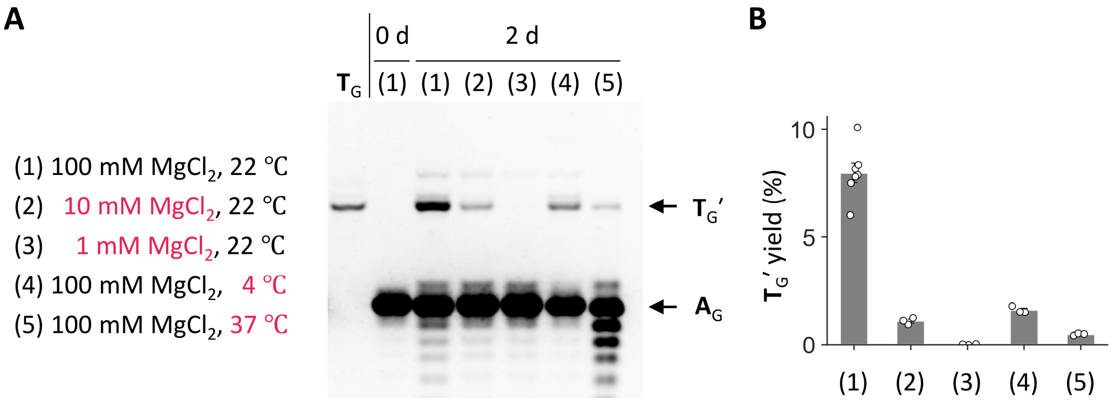

Fig. S12. Autocatalytic reproduction of T_G' in different environments. (A) Incubation of FAM-labeled $\mathbf{A}_G$, $\mathbf{B}_S$, and $\mathbf{T}_G'$ (20 μM each) for 2 d in different $MgCl_2$ concentrations or at different temperatures, as described in the panel. The reactions were analyzed by 20% denaturing PAGE. Pure $\mathbf{T}_G$ was run in parallel as a size control. (B) Yields of $\mathbf{T}_G'$ quantified from fluorescence intensities. Data for (1) are the same as in Fig. 4D. Error bars indicate standard errors ($n = 3$).

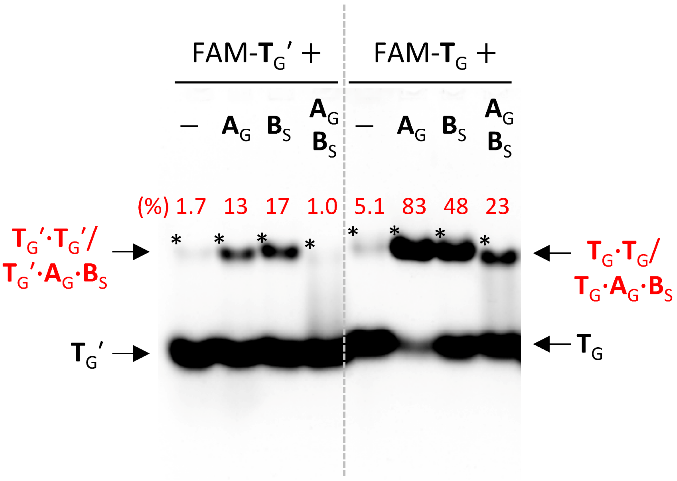

Fig. S13. The effect of 2′-5′ phosphodiester linkages on higher-order RNA complex formation. (A) Native PAGE analysis of RNA mixtures containing either **T**G′ (with a 2′-5′ linkage) or **T**G (containing only 3′-5′ linkages). Various combinations of RNAs (20 μM each) containing FAM-labeled **T**G′ or **T**G were incubated in 100 mM $MgCl_2$ at 22 °C for 3 h, and then immediately subjected to 20% native PAGE in 20 mM $MgCl_2$ at 22 °C. Percentages of products of interest (indicated with asterisks) were shown above the product bands.

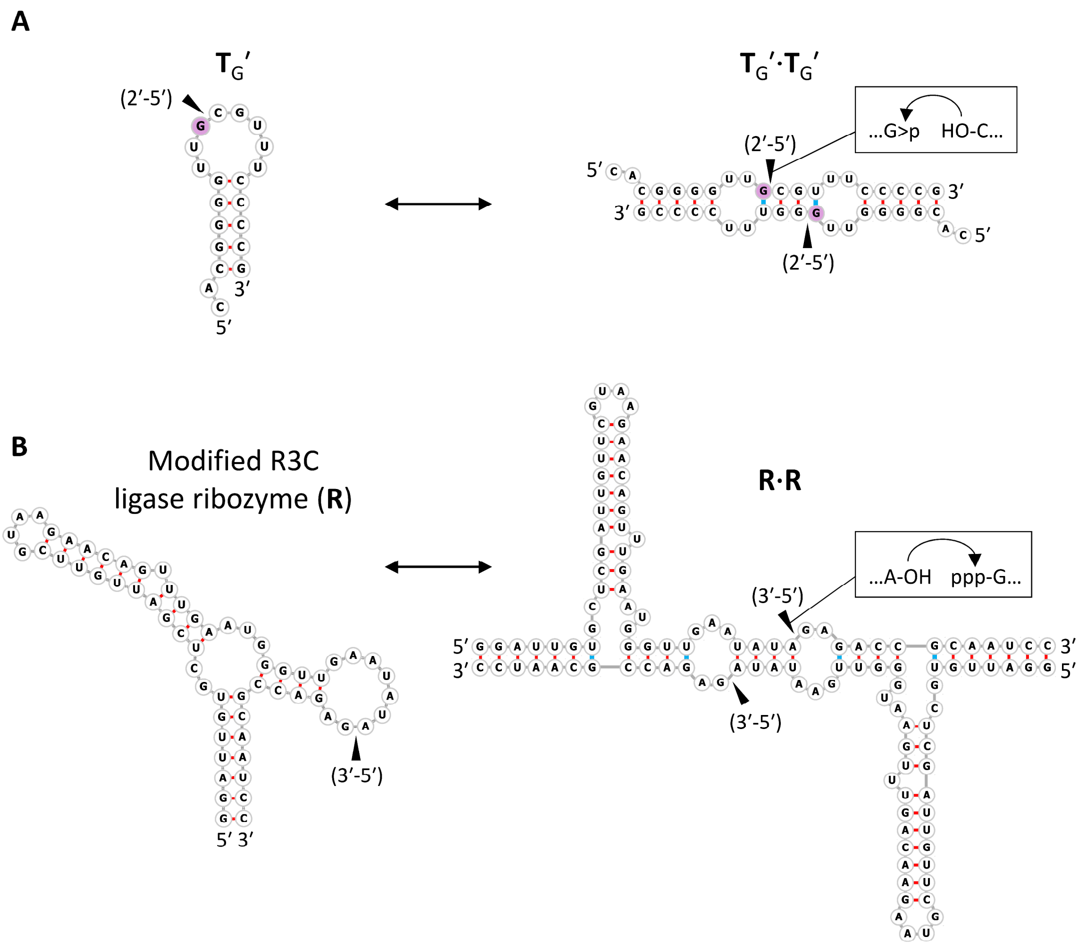

Fig. S14. Predicted secondary structures of **T**G′ and a self-reproducing ribozyme constructed previously.[8] The monomer and dimer structures of **T**G′ (A) and the ribozyme (B) are displayed. The dimeric structure of the ribozyme was predicted based on the previous study.[8] For dimers, G•U wobble pairs at the template regions were highlighted in cyan. Black arrowheads indicate ligation junctions.

Table S1. List of oligonucleotides.

Name	DNA/RNA	Sequence (from 5' end to 3' end)
N_{20}	RNA	NNNNNNNNNNNNNNNNNNNN
N_{20}>p	RNA	NNNNNNNNNNNNNNNNNNNN>p
f1-1	RNA	6-FAM-CACGGGGUUCGUUUCCCCGCACCUCCACC
**A**	RNA	6-FAM-CACGGGGUU>p
$\mathbf{A}_{N11}$	RNA	6-FAM (or OH)-CACGGGGUUNNNNNNNNNNN
$\mathbf{A}_G$	RNA	6-FAM (or OH)-CACGGGGUUG>p (or -p)
$\mathbf{A}_A$	RNA	6-FAM-CACGGGGUUA>p
$\mathbf{A}_U$	RNA	6-FAM-CACGGGGUUU>p
$\mathbf{A}_C$	RNA	6-FAM-CACGGGGUUC>p
**B**	RNA	CGUUUCCCCGCACCUCCACC
$\mathbf{B}_S$	RNA	CGUUUCCCCG
**T**	RNA	CACGGGGUUCGUUUCCCCG
$\mathbf{T}_G$	RNA	OH (or 6-FAM)-CACGGGGUUGCGUUUCCCCG
$\mathbf{T}_G$′	RNA	OH (or 6-FAM)-CACGGGGUUG2′-5′CGUUUCCCCG
$\mathbf{T}_A$	RNA	CACGGGGUUACGUUUCCCCG
$\mathbf{T}_U$	RNA	CACGGGGUUUCGUUUCCCCG
$\mathbf{T}_C$	RNA	CACGGGGUUCCGUUUCCCCG
Primer 1	DNA	GTGACTGGAGTTCAGACGTGTGCTCTTCCGATCTTT TTTTTTTTTTTTGGTG
Primer 2	DNA	CTCTTTCCCTACACGACGCTCTTCCGATCTGGGCAC
Primer 3	DNA	GTGACTGGAGTTCAGACGTGTGC
Primer 4	DNA	CGGCGACCACCGAGATCTACACTTCAGGTCACACT CTTTCCCTACACGACGCTCTTCCGATCTGGGCAC

Nucleotides and linkages added to A or T are shown in red.

References

1 H. Mutschler and P. Holliger, *J. Am. Chem. Soc.*, 2014, **136**, 5193–5196.

2 M. Martin, *EMBnet. J.*, 2011, **17**, 10–12.

3 H. Mutschler, A. I. Taylor, A. Lightowlers, G. Houlihan, M. Abramov, P. Herdewijn and P. Holliger, *Elife*, 2018, **7**, e43022.

4 R. Lorenz, S. H. Bernhart, C. Höner zu Siederdissen, H. Tafer, C. Flamm, P. F. Stadler and I. L. Hofacker, *Algorithms Mol. Biol.*, 2011, **6**, 26.

5 K. Katoh, J. Rozewicki and K. D. Yamada, *Brief. Bioinform.*, 2018, **20**, 1160–1166.

6 A. Tareen and J. B. Kinney, *Bioinformatics*, 2020, **36**, 2272–2274.

7 P. Kerpedjiev, S. Hammer and I. L. Hofacker, *Bioinformatics*, 2015, **31**, 3377–3379.

8 N. Paul and G. F. Joyce, *Proc. Natl. Acad. Sci. U. S. A.*, 2002, **99**, 12733–12740.
